# Engineering TATA-binding protein Spt15 to improve ethanol tolerance and production in *Kluyveromyces marxianus*

**DOI:** 10.1186/s13068-018-1206-9

**Published:** 2018-07-24

**Authors:** Pengsong Li, Xiaofen Fu, Shizhong Li, Lei Zhang

**Affiliations:** Institute of New Energy Technology, MOST-USDA Joint Research Center for Biofuels, Beijing Engineering Research Center for Biofuels, Tsinghua University, Beijing, 100084 China

**Keywords:** Global transcription machinery engineering (gTME), TATA-binding protein, Spt15, *Kluyveromyces marxianus*, Ethanol tolerance, Fermentation

## Abstract

**Background:**

Low ethanol tolerance of *Kluyveromyces marxianus* limits its application in high-temperature ethanol fermentation. As a complex phenotype, ethanol tolerance involves synergistic actions of many genes that are widely distributed throughout the genome, thereby being difficult to engineer. TATA-binding protein is the most common target of global transcription machinery engineering for improvement of complex phenotypes.

**Results:**

A random mutagenesis library of *K. marxianus* TATA-binding protein Spt15 was constructed and subjected to screening under ethanol stress. Two mutant strains with improved ethanol tolerance were identified, one of which (denoted as M2) exhibited increased ethanol productivity. The mutant of Spt15 in strain M2 (denoted as Spt15-M2) has a single amino acid substitution at position 31 (Lys → Glu). RNA-Seq-based transcriptomic analysis revealed cellular transcription profile changes resulting from Spt15-M2. Spt15-M2 caused changes in transcriptional level of most of the genes in the central carbon metabolism network. Compared with control strain, 444 differentially expressed genes (DEGs) were identified in strain M2 (fold change > 2, *P*_adj_ < 0.05), including 48 up-regulated and 396 down-regulated. The up-regulated DEGs are involved in amino acid transport, long-chain fatty acid biosynthesis and MAPK signaling pathway, while the down-regulated DEGs are related to ribosome biogenesis, translation and protein synthesis. Five candidate genes (*GAP1*, *GNP1*, *FAR1*, *STE2* and *TEC1*), which were found to be up-regulated in M2 strain, were overexpressed for a gain-of-function assay. However, the overexpression of no single gene helped improve ethanol tolerance as *SPT15*-M2 did.

**Conclusions:**

This work demonstrates that ethanol tolerance of *K. marxianus* can be improved by engineering its TATA-binding protein. A single amino acid substitution (K31E) of TATA-binding protein Spt15 is able to bring differential expression of hundreds of genes that acted as an interconnected network for the phenotype of ethanol tolerance. Future perspectives of this technique in *K. marxianus* were discussed.

**Electronic supplementary material:**

The online version of this article (10.1186/s13068-018-1206-9) contains supplementary material, which is available to authorized users.

## Background

Bioethanol is becoming increasingly important due to the decreasing fossil energy resources and global warming claims. To produce bioethanol costly and effectively, we have developed a novel advanced solid-state fermentation (ASSF) technology to produce ethanol using crushed sweet sorghum stalks, which is equipped with an optimized and redesigned rotary drum fermenter and a proprietary yeast strain [1–4]. However, low efficiencies of mass and heat transfer limit the industrial application of solid-state fermentation (SSF) [1, 5]. The rotary drum fermenter of the ASSF system improves the mass and heat transfer efficiencies and can thereby increase the ethanol productivity from sweet sorghum to a great extent [6], first demonstrating that SSF can be applied at industrial scale for ethanol production [2]. Nevertheless, there is still substantial reaction heat from metabolic activities of microorganisms trapped within the solid matrix, which always makes the temperature of SSF system over 40 °C, especially for the ASSF-driven ethanol plants resided in tropical and subtropical regions.

Thermotolerant yeast *Kluyveromyces marxianus* can grow well at temperatures as high as 45–52 °C and can efficiently produce ethanol at temperatures between 38 and 45 °C [7, 8], which makes it a good candidate for ethanol fermentation at elevated temperatures. In addition, *K. marxianus* can utilize various pentoses and hexoses as single carbon sources [9], thereby becoming an ideal host for cellulosic ethanol production by simultaneous saccharification and fermentation [10]. Like other pentose-utilizing yeast species, however, *K. marxianus* has lower ethanol tolerance compared with *Saccharomyces cerevisiae* [11], which limits its application in ethanol fermentation.

Ethanol tolerance is thought to be associated with multiple genes that are widely distributed throughout the genome. Although significant efforts have been made to study ethanol tolerance in past decades, its mechanisms have not been well known yet [12]. Therefore, overexpression or deletion of a single gene can hardly reach an ideal phenotype due to the complexity of metabolic landscapes [13, 14]. To address this problem, Alper et al. [15] developed an approach termed “global transcription machinery engineering (gTME)”, which has been widely used to evolve the desired phenotypes in recent years [16–22]. Through mutagenesis (via error-prone PCR mutations) of key proteins regulating the global transcriptome, gTME allows for global perturbations of the transcriptome so that improved complex phenotypes can be elicited quickly and effectively. The most commonly targeted component of yeast for gTME is TATA-binding protein (TBP) Spt15 [15, 23–25], which is one of the components of the general factor RNA polymerase II (RNA Pol II) transcription factor D (TFIID). *K. marxianus* Spt15 has been engineered to improve acid resistance and 3-hydroxypropionate production [26], demonstrating the applicability of this strategy to *K. marxianus*. Thus, we chose *K. marxianus* Spt15 as the target protein in this study for its putative ability to control as much as 90% of the yeast transcriptome by its association with the general transcription complex TFIID [27, 28]. The *SPT15* gene was subjected to error-prone PCR and cloned into an expression vector. Pooled recombinant plasmids were then transformed into *K. marxianus* to construct a random mutagenesis library. Then the library was subjected to screening under ethanol stress. Two mutant strains with improved ethanol tolerance were identified using this method, one of which (denoted as M2) showed increased ethanol productivity. Then strain M2 was subjected to RNA-Seq-based transcriptomic analysis to investigate the perturbations of transcriptome resulting from Spt15-M2.

## Results and discussion

### Random mutagenesis library construction

A random mutagenesis library of *SPT15* containing 10^5^ *E. coli* clones was constructed by transformation of *E. coli* TOP10 with the pooled plasmids containing *SPT15*-*mt*-*P2A*-*GFP* cassettes (Additional file 1: Fig. S1). The P2A peptide between a Spt15 mutant and a GFP functions as a *cis*-acting hydrolase element, mediating “cleavage” between the two proteins so that they can be generated separately from one open reading frame [8, 29]. This method can minimize the influence of GFP because a Spt15 mutant can exercise its function as a single protein instead of a GFP-fused one. To determine the mutation frequency of the mutagenesis library, 20 *E. coli* clones were randomly picked and the *SPT15* mutants were sequenced using SEQ-F as primer. The nucleotide changes of the *SPT15* mutants in the 20 randomly picked clones were counted. According to the statistical result, the average mutation frequency was 2.3 nucleotide changes per gene (Additional file 1: Table S1). Since the mutation frequencies of two to seven nucleotide changes per gene are commonly employed in previous studies [30–32], 2.3 nucleotide changes per gene is thought to be proper in the present study. Then all the 10^5^ clones were mixed and the pooled plasmids were extracted. A random mutagenesis library containing 10^5^ *K. marxianus* clones was then constructed by transformation of DMKU3-1042 with the pooled plasmids. The successful expression of the *SPT15* mutants was confirmed by observing the fluorescence of GFP. The result of fluorescence microscopy indicates that the library was successfully constructed (Additional file 1: Fig. S2). The efficiency of P2A cleavage in the strain harboring *SPT15*-*P2A*-*GFP* cassette and the random mutagenesis library was examined using semiquantitative western blotting analysis. When anti-GFP antibody was used as primary antibody in western blotting, both GFP and the uncleaved fusion of Spt15-P2A-GFP were detected (Additional file 1: Fig. S3). The calculated cleavage efficiency was 88.66–93.20% (Additional file 1: Fig. S3), demonstrating that P2A cleavage is efficient in *K. marxianus*.

### Mutant screening under ethanol stress

**Fig. 1:**
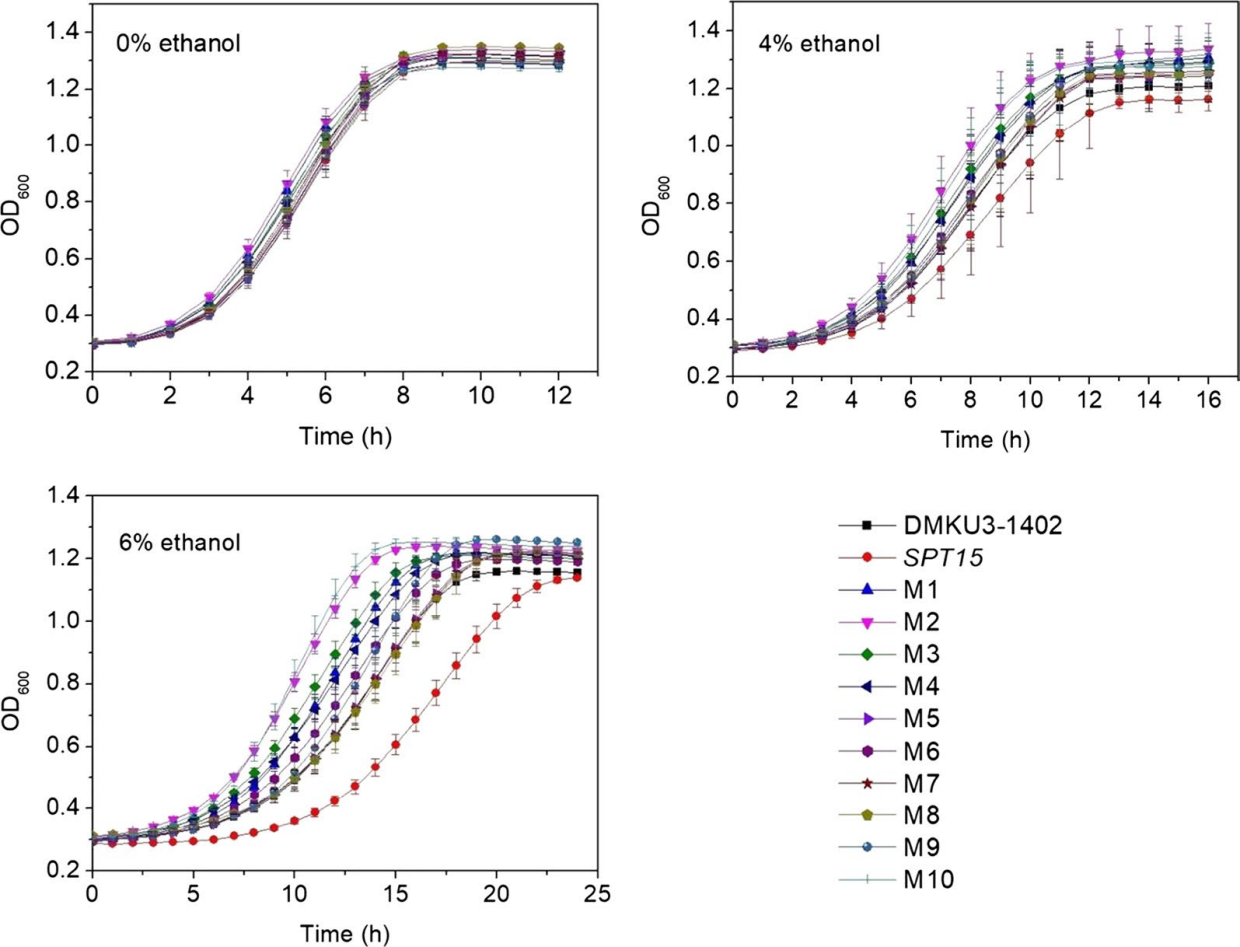
Growth curves of mutant strains at 42 °C under different concentrations of ethanol. Overnight cultures were diluted with YPD medium to reach an initial OD_600_ of 0.20. These cell suspensions were aliquoted in triplicates into a sterile 96-well plate with 200 μL in each well and incubated at 42 °C in a microplate reader to measure the growth curves. Values are means and standard deviations (*n* = 3)

**Fig. 2:**
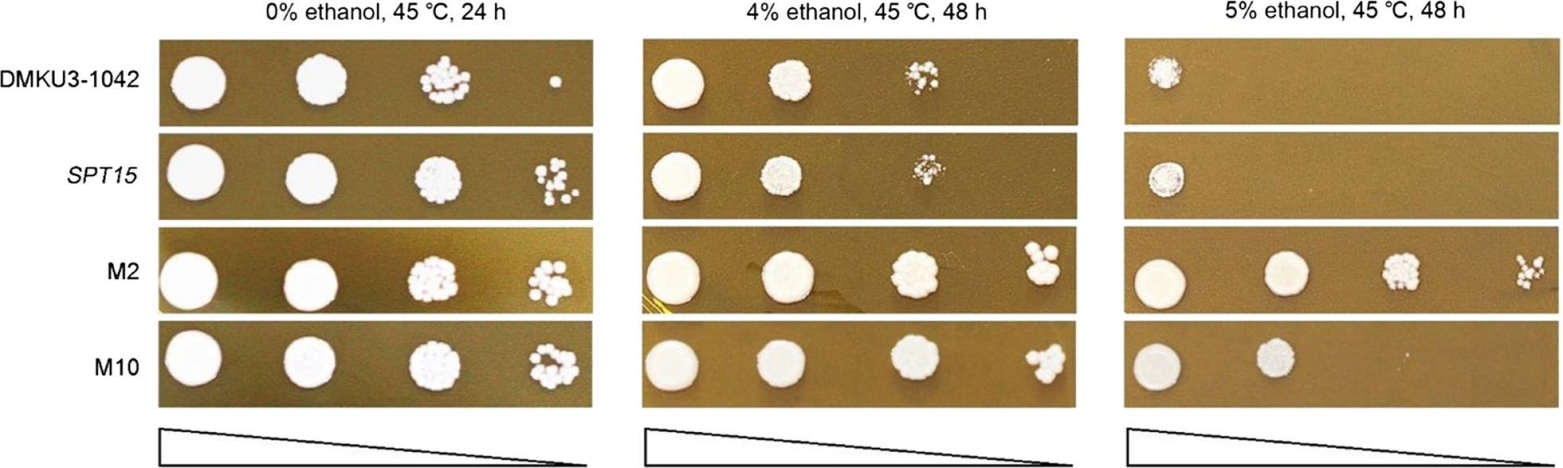
Spotting test of mutant strains M2 and M10 at 45 °C under different concentrations of ethanol. 2 μL cell suspensions of each strain with OD_600_ of 0.20 and serial dilutions of 10^−1^ to 10^−3^ were spotted onto YPD agar medium and then incubated at 45 °C. *SPT15* stands for the strain overexpressing wild-type *SPT15*

The random mutagenesis library of *K. marxianus* was cultured in 200 mL YPD medium supplemented with elevated ethanol concentration [from 2 to 5% (v/v)] at 45 °C. After five successive subcultures, the enriched cell culture was diluted and spread onto YPD plates and ten individual colonies were randomly picked. The growth performances of the ten mutant strains were tested under ethanol stress in 96-well plates. The growth curve data were fitted with logistic model and the results show that all sets of data fitted the model well (*R*^2^ > 0.99) (Additional file 2). Two strains M2 and M10 were found to grow faster than other strains under ethanol stress and their advantages were most obvious under the condition of 6% ethanol (Fig. 1). Then the first-order derivative functions of the growth curves were calculated to show the variations of growth speed as a function of time. The maximum time derivatives of M2’s and M10’s growth curves were higher and appeared earlier than those of DMKU3-1042 and the strain overexpressing wild-type *SPT15* in the presence of ethanol. Especially in the presence of 6% ethanol, the maximum time derivatives of M2’s and M10’s growth curves were 0.121 and 0.108/h, respectively, both appeared at around 8 h after inoculation; while those of DMKU3-1042 (0.089/h) and the strain overexpressing wild-type *SPT15* (0.070/h) appeared about 3 and 8 h later, respectively (Additional file 1: Fig. S4). Further spotting tests show that M2 and M10 exhibited better survival than both DMKU3-1042 and the strain overexpressing wild-type *SPT15* on YPD agar plates containing ethanol at 45 °C (Fig. 2). Despite similar growth performance to wild-type DMKU3-1042 when no ethanol added, strain overexpressing wild-type *SPT15* grow more poorly under conditions involving ethanol (Fig. 1). This phenomenon can be attributed to the so-called “plasmid burden”: the replication and maintenance of plasmid DNA causes burdens on yeast cells, leading to a reduced growth rate [33]. To assess the effect of plasmid burden in this study, we conducted another growth curve assay in which wild-type DMKU3-1042 and strains harboring a blank plasmid and a plasmid containing wild-type *SPT15* were used. As shown in Additional file 1: Fig. S5, both of the plasmid-harboring strains exhibited poorer growth compared to wild-type DMKU3-1402, especially under conditions involving ethanol. This finding indicates that the plasmid burden did affect cell growth in this study. Thus, to eliminate the influence of plasmid burden, the strain overexpressing wild-type *SPT15* was used as control strain in the following experiments.

### Ethanol fermentation of the mutant strains

**Fig. 3:**
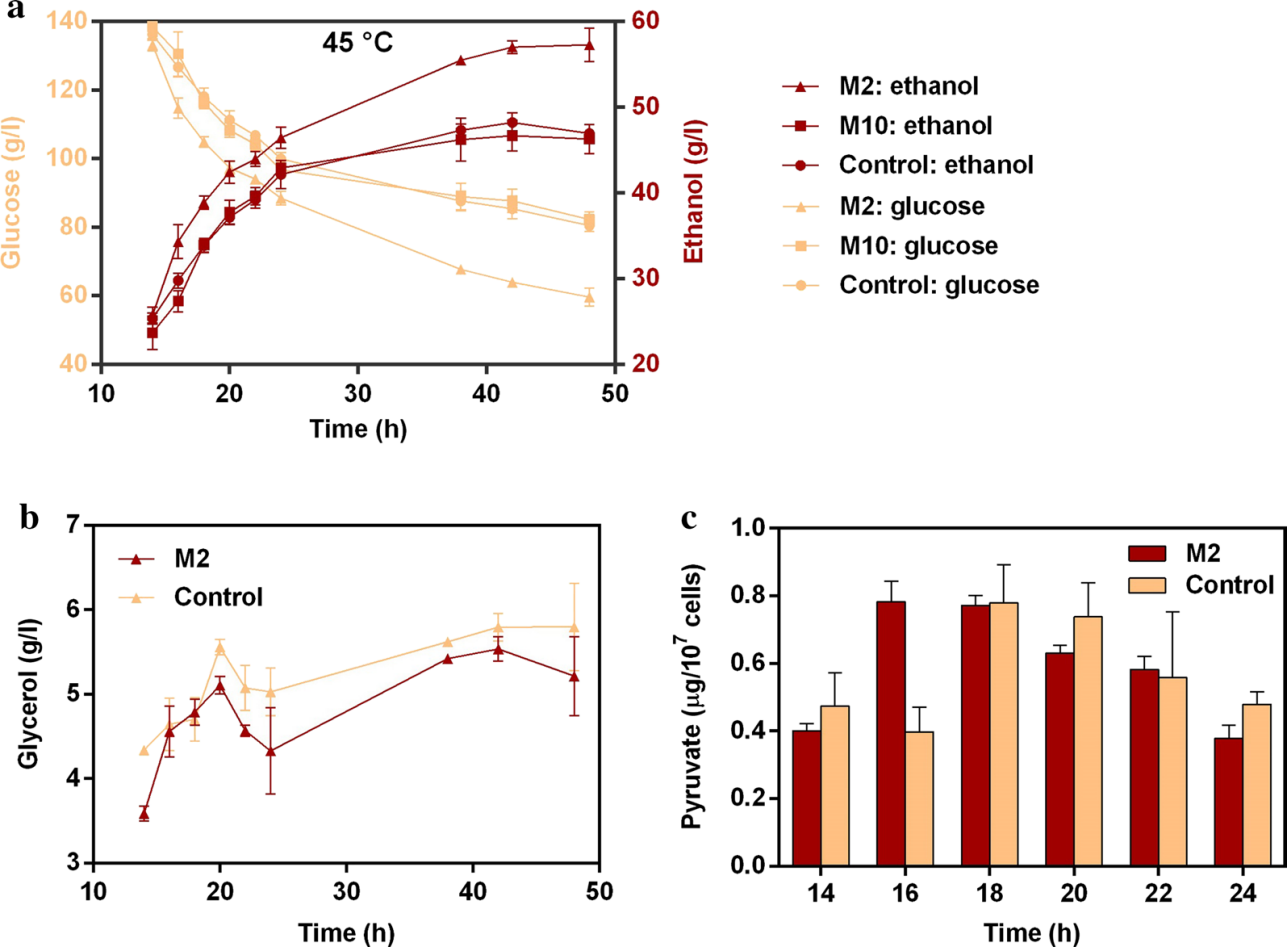
Fermentation results: concentrations of **a** ethanol and residual glucose, **b** glycerol, and **c** intracellular content of pyruvate during batch ethanol fermentation. Data were collected from batch fermentation experiments conducted in sealed 100-mL serum bottles at 45 °C with three biological replicates. Control: the strain overexpressing wild-type *SPT15*. Values are means and standard deviations (*n* = 3)

Batch fermentation experiments were conducted at 45 °C to assess the ethanol fermentation performance of the mutant strains M2 and M10. According to the fermentation results, strain M2 exhibited better fermentation performance compared with M10 and control strain (Fig. 3a). It should be noted that the fermentation processes of all the strains were incomplete due to the inhibition of accumulated ethanol, with about 60–80 g/L glucose left at the end of fermentation. We defined “ethanol inhibition concentration” (EIC) as the ethanol concentration when fermentation stopped due to ethanol inhibition. Accordingly, the EIC of strain M2 is about 57 g/L, while those of M10 and control strain are about 46 and 47 g/L, respectively. After 24 h of fermentation, strain M2 produced 46.47 ± 1.25 g/L ethanol, which was 8.30 and 10.28% higher than that produced by M10 and control strain, respectively; while after 48 h of fermentation, strain M2 produced 57.29 ± 1.96 g/L ethanol, which was 23.74 and 22.05% higher than that produced by M10 and control strain, respectively. Then we reconstructed two strains without P2A-GFP, which overexpress wild-type *SPT15* and *SPT15*-M2, respectively. The fermentation performance of these two strains was further tested. The results show that the strain overexpressing *SPT15*-M2 mutant still maintained the advantage in ethanol fermentation (Additional file 1: Fig. S6). These results indicate that the mutation in *SPT15*-M2 confers the yeast higher ethanol tolerance, enabling it to produce higher concentration of ethanol during batch fermentation.

### Mutation site identification

The plasmid containing the *SPT15*-M2 mutant was extracted from strain M2 and was sequenced to identify the mutation sites. According to the sequencing result, *SPT15*-M2 has a single base substitution, resulting in a single amino acid substitution at position 31 (Lys → Glu). Like other TBPs, Spt15 has a phylogenetically conserved C-terminal region which is made up of ~ 180 amino acids and a highly diverged species-specific N-terminal region which varies in both length and sequence [34]. The conserved C-terminal region recognizes the TATA box, with an 8-bp consensus sequence TATA(A/T)A(A/T)(A/G) [34, 35]. Although the nonconserved N-terminal region is largely unnecessary for transcription in certain yeast strains [35], Zhou et al. [36] have found that the acidic region just N-terminal to the conserved region is required for normal growth and transcription control in most yeast strains. In addition, previous studies have found that the N-terminal region of human TBP inhibits TATA binding by the C-terminal region of TBP on both pol III- [37] and pol II-dependent promoters [38]. It is suggested that human TBP binds to the TATA box through a two-step process, including an initial binding of TBP to the TATA box without bending the DNA and followed by a slow transition into a more stable bent TATA–TBP complex. The nonconserved N-terminal region inhibits formation of the bent TBP–TATA box complex and promotes formation of the unbent TBP–TATA box complex [38]. To the best of our knowledge, no similar function of yeast TBP’s N-terminal region has been reported. Considering the phylogenetic conservation of TBPs, however, the N-terminal region of yeast TBP may have a similar function with that of human TBP. Thus, the K31E substitution in this study may change the inhibition of Spt15’s N-terminal region to the formation of TATA–Spt15 complex, and then the efficiency of transcription initiation may be changed accordingly.

### Spt15-M2 resulted in transcriptome perturbations

To investigate the perturbations of transcriptome resulting from Spt15-M2, strain M2 during ethanol fermentation at 45 °C was subjected to RNA-Seq-based transcriptomic analysis with three biological replicates.

**Fig. 4:**
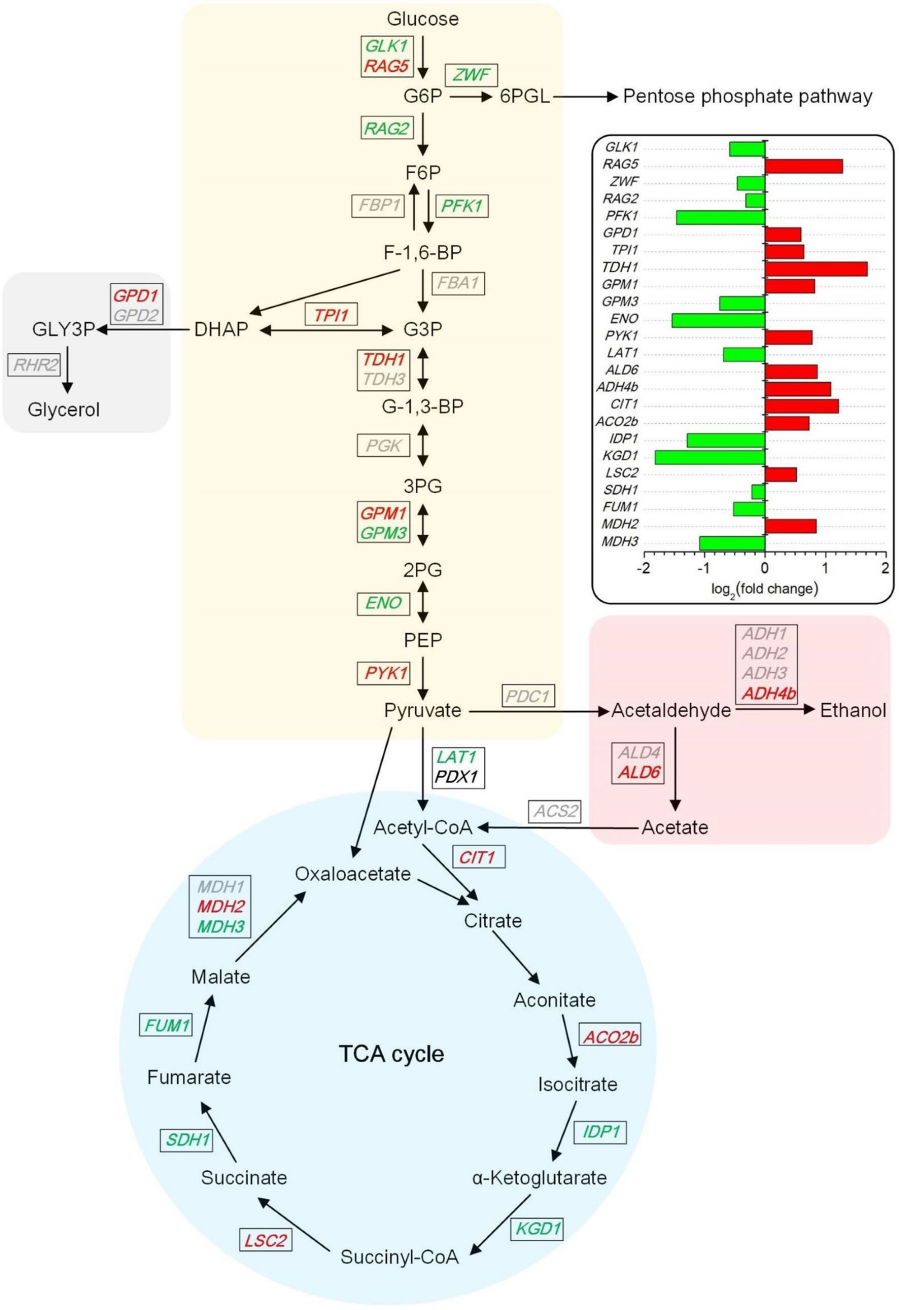
The central carbon metabolic network of *K*. *marxianus*. Red colored: significantly up-regulated genes for M2 vs *SPT15*; green colored: significantly down-regulated genes for M2 vs *SPT15*; grey colored: genes with no significant fold change. The log_2_(fold change) values of the genes are illustrated at the top-right corner. *SPT15* stands for the strain overexpressing wild-type *SPT15*

We first reconstructed the central carbon metabolic network based on the RPKM values of gene involved in this network (Fig. 4 and Additional file 3). The results show that Spt15-M2 caused changes in transcriptional level of most of the genes in the central carbon metabolism network. Glucose is phosphorylated by hexose–glucose kinase after uptake, and then enters the glycolytic pathway. The hexokinase gene *RAG5* in strain M2 had more than twofold up-regulation compared with that in control strain, while the glucokinase gene *GLK1* was slightly down-regulated. Considering that the hexokinase of *K. marxianus* can phosphorylate both glucose and fructose [39], the up-regulation of *RAG5* in strain M2 might promote its glucose assimilation ability. The glucose-6-phosphate dehydrogenase gene *ZWF* was down-regulated in strain M2, which might decrease the carbon flux through the pentose phosphate pathway (PPP) [40]. *PFK1*, which encodes 6-phosphofructokinase subunit alpha, was down-regulated by 2.5-fold in strain M2. However, a previous study has found that reduced *PFK1* expression had no significant effect on growth rate, glucose consumption or ethanol production [41]. *TPI1* and *GPD1* were up-regulated in strain M2. These two genes encode enzymes in the pathway of glycerol formation, triose phosphate isomerase and glycerol-3-phosphate dehydrogenase, respectively. Glycerol is considered as a byproduct for ethanol production, serving as an essential electron sink for nicotinamide adenine dinucleotide (NADH) generated in biosynthesis during anaerobic growth [42]. Thus, yeast cells produce glycerol as an alternative means of NAD^+^ regeneration to deal with the unbalanced cellular NAD^+^/NADH ratio caused by ethanol exposure [43]. Surprisingly, although the transcription levels of *TPI1* and *GPD1* were higher in strain M2 compared with control strain, HPLC results show that strain M2 produced slightly less glycerol than control strain did (Fig. 3b), indicating its higher carbon flux towards ethanol formation. Pyruvate, which is an important intermediate metabolite of the central carbon metabolism, determines the carbon fluxes to ethanol production or to the tricarboxylic acid (TCA) cycle. According to the results of pyruvate assay, the maximum intracellular content of pyruvate in M2 strain appeared at 16 h, while that in the control strain appeared 2 h later (Fig. 3c). This finding indicates that M2 strain accumulated more pyruvate via glycolysis during early fermentation stage, which in turn helped to increase fermentation rate. The pyruvate kinase Pyk1 catalyzes phosphoenolpyruvate (PEP) into pyruvate and is regarded as the rate-limiting enzyme [44]. The pyruvate kinase gene *PYK1* was found up-regulated by 1.7-fold in strain M2; which might help produce pyruvate for ethanol fermentation.

*CIT1*, encoding the mitochondrial citrate synthase Cit1, was found up-regulated by 2.3-fold in strain M2. Cit1 catalyzes the first reaction of the TCA cycle which is condensation of acetyl-CoA and oxaloacetate to form citrate. Thus, Cit1 functions as a rate-limiting enzyme of the TCA cycle [45]. Under anaerobic conditions, the TCA cycle can work as a reducing cycle, reducing the excess NADH [46]. Furthermore, the activity of TCA pathway is also thought to be maintained for primary fuel biosynthetic reactions supplying cells with oxaloacetate and 2-oxoglutarate, the precursors of aspartate and glutamate [47]. *ALD6*, encoding aldehyde dehydrogenase that catalyzes acetaldehyde into acetic acid, was also up-regulated in strain M2. It is assumed that yeast cells produce acetic acid to regenerate reducing equivalents such as nicotinamide adenine dinucleotide phosphate (NADPH) in the cytoplasm [48], which can neutralize reactive oxygen species (ROS) generated at high temperatures [49]. The alcohol dehydrogenase Adh4 gene *ADH4b* in strain M2 was up-regulated by more than twofold. This gene is thought to be involved in the degradation of ethanol and thereby contribute to ethanol detoxification to ensure cell survival [50, 51]. Hence, the enhanced transcription of *ADH4b* in strain M2 might confer it higher ethanol tolerance.

**Fig. 5:**
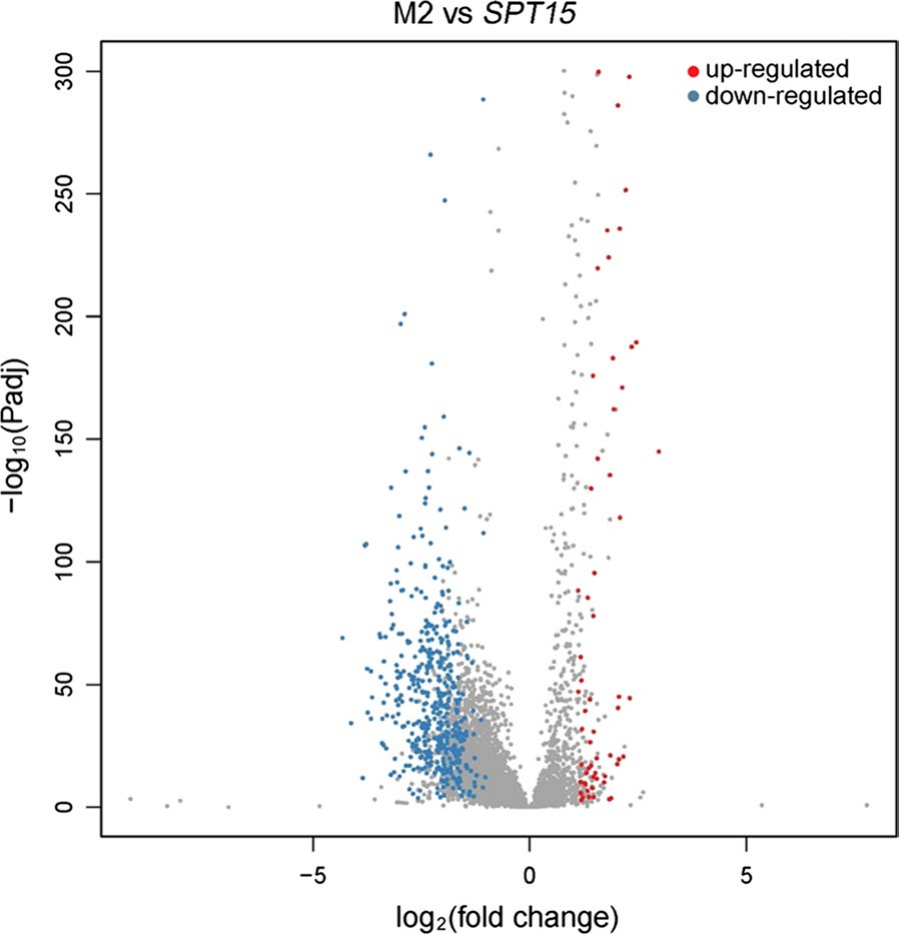
Volcano plots of differentially expressed genes (DEGs) for M2 vs *SPT15*. Genes with adjusted *P* values (*Padj*) less than 0.05 and log_2_(fold change) values greater than 1 were assigned as differentially expressed. S*PT15* stands for the strain overexpressing wild-type *SPT15*

To further explore the transcriptome perturbations caused by Spt15-M2, we conducted differential expression analysis based on the RNA-Seq data. Strain M2 was identified to have 444 differentially expressed genes (DEGs) (including 48 up-regulated and 396 down-regulated) compared with control strain (Fig. 5 and Additional file 4). Gene Ontology (GO) and KEGG enrichment analyses were conducted to identify the functions of DEGs. The results of GO enrichment show that biological processes related to amino acid transport (such as GO: 0055085, GO: 0003333, GO: 0035524 and GO: 0006865) and long-chain fatty acid biosynthesis (GO: 0042759) were enriched in the up-regulated DEGs (Additional file 5). Ethanol can enhance the passive proton flux through the yeast plasma membrane, leading to depolarization of membrane potential and inhibition of nutrient uptake (such as amino acids and ammonium) [52, 53]. The exposure of yeast cells to ethanol also fluidizes the plasma membrane [54, 55]. It has been reported that the incorporation of the supplementary amino acids into plasma membrane can lead to enhanced ability for plasma membrane to efficiently counteract the fluidizing effect of ethanol when subjected to ethanol stress [56, 57]. Therefore, the up-regulation of genes related to amino acid transport in strain M2 might reduce ethanol’s fluidizing effect and inhibition on amino acid uptake, resulting in higher ethanol tolerance. In addition, long-chain fatty acids in plasma membrane also play a critical role in ethanol resistance in *S. cerevisiae* [53, 58]. Among the up-regulated DEGs, *FAS1* and *ACC1*, which were also found up-regulated in *S. cerevisiae* strain expressing *K. marxianus MSN2* in our previous study [8], are related to long-chain fatty acid biosynthesis. Thus, the up-regulation of *FAS1* and *ACC1* in strain M2 might improve ethanol tolerance by enhancing long-chain fatty acid biosynthesis. According to the KEGG enrichment result, MAPK signaling pathway was enriched in the up-regulated DEGs in strain M2. *STE2*, *FAR1* and *TEC1* are involved in pheromone-induced MAPK signaling pathway [59, 60]. Previous studies also found that genes involved in pheromone pathways were up-regulated specifically in haploid strains [61, 62], indicating that their mating system was robust to environmental variation [62]. Therefore, the up-regulation of genes associated with pheromone pathway in strain M2 might confer the cells more robust mating to deal with ethanol stress. *K. marxianus* was reported to be homothallic [63, 64]. Although homothallism is a form of mating that produces minimal genetic variability, homothallic mating in fungi is thought to be an adaptation for surviving stressful conditions because promoted homologous recombination can repair DNA damages caused by a stressful environment [65]. Regarding the enrichment analyses of the down-regulated DEGs, the biological processes related to translation and protein synthesis were enriched, including ribosome biogenesis, rRNA processing, tRNA processing, and so on (Additional files 5 and 6). A similar result was given by the interaction analysis of the identified DEGs. According to the result of interaction analysis, the interactions between the DEGs were clustered into several groups, the largest among which includes a number of closely interrelated genes related to ribosome biogenesis and rRNA processing. Other groups include genes involved in ncRNA transcription, mRNA metabolic process, Golgi vesicle transport, posttranslational protein targeting to membrane, ubiquitin-dependent protein catabolic process, dicarboxylic acid metabolism and mitochondrial membrane organization, respectively (Table 1 and Additional file 1: Fig. S7). Most of these genes were also found repressed in response to ethanol stress by previous studies [66–68]. In a previous study, GO analysis revealed the following GO terms to be overrepresented in the DEGs in an ethanol-tolerant *S. cerevisiae* strain expressing a *SPT15* mutant: oxidoreductase activity, cytoplasmic proteins and enzymes, amino acid and derivative metabolism, vitamin metabolism, and electron transport [15]. In another study in which RNA polymerase II subunit Rpb7 was engineered to improve ethanol tolerance in *S. cerevisiae*, genes involved in energy metabolism, oxidative stress response, long-chain fatty acid metabolism and sterol synthesis were found up-regulated and genes related to helicase activity, DNA repair, ribosome assembly were down-regulated in the ethanol-tolerant strain [22]. By comparing the results of transcriptomic analysis in the present study with those in the previous studies, we found that enhanced long-chain fatty acid biosynthesis and repressed ribosome assembly were elicited in ethanol-tolerant strains of both *K. marxianus* and *S. cerevisiae* [15, 22], suggesting their conserved mechanism of ethanol tolerance. Intriguingly, up-regulated expression of gene related to amino acid transport and MAPK signaling pathway was only found in this study, which provides a new insight into the mechanism of ethanol tolerance.

**Table 1:** List of interacted DEGs for M2 vs *SPT15*. *SPT15* stands for the strain overexpressing wild-type *SPT15*

Category	Gene list
Ribosome biogenesis and rRNA processing	*BMS1*, *CBF5*, *CGR1*, *CIC1*, *DBP3*, *DBP7*, *DHR2*, *DRS1*, *EBP2*, *ECM16*, *EFG1*, *ENP1*, *FAL1*, *FCF2*, *GAR1*, *IMP4*, *IPI3*, *KRI1*, *MAK11*, *MAK16*, *MDN1*, *MTR4*, *NAN1*, *NIP7*, *NOC4*, *NOP12*, *NOP16*, *NOP53*, *NOP58*, *NOP7*, *NOP8*, *NOP9*, *NSA1*, *NUG1*, *PNO1*, *PWP1*, *PXR1*, *RCL1*, *RIO2*, *RLP7*, *ROK1*, *RRB1*, *RRP14*, *RRP3*, *RRP4*, *RRP42*, *RRP43*, *RRP45*, *RRP46*, *RRP8*, *RRS1*, *SAS10*, *SGD1*, *SKI6*, *SLX9*, *SPB4*, *SQT1*, *SSF1*, *UTP10*, *UTP18*, *UTP25*, *UTP30*, *UTP4*, *UTP9*, *YVH1*, etc.
ncRNA transcription	*PZF1*, *RPC11*, *RPC25*, *RPC37*, *RPC53*, *RPO31*, *TFC1*, etc.
mRNA metabolic process	*CUS1*, *NAM8*, *NPL3*, *PRP42*, *RPB7*, *SPT4*, etc.
Golgi vesicle transport	*EMP47*, *ERP1*, *ERV25*, *ERV29*, *SEC13*, *SEC18*, *SEC22*, *TLG2*, *TRS33*, *UFE1*, etc.
Posttranslational protein targeting to membrane	*GET3*, *GET4*, *SBH1*, *SEC61*, *SEC62*, *SEC72*, *SGT2*, etc.
Ubiquitin-dependent protein catabolic process	*DOA1*, *PRE2*, *PRE3*, *PRE7*, *PUP1*, *PUP3*, *RPN5*, *RPT3*, *RPT4*, *UBP6*, etc.
Dicarboxylic acid metabolism	*AAT1*, *FOL1*, *GDH3*, *KGD1*, *MIS1*, etc.
Mitochondrial membrane organization	*TAZ1*, *TOM20*, *TOM70*, etc.

To explore whether overexpression of an individual gene can lead to improved ethanol tolerance, five candidate DEGs (*GAP1*, *GNP1*, *FAR1*, *STE2* and *TEC1*), which were identified to be up-regulated in M2 strain, were overexpressed for a gain-of-function assay. Among these five genes, *GAP1* and *GNP1* are involved in amino acid transport, while *FAR1*, *STE2*, and *TEC1* are related to MAPK signaling pathway. We found that the overexpression of no single gene helped improve ethanol tolerance as *SPT15*-M2 did (Additional file 1: Fig. S8). This finding is consistent with another study [15]. Previous studies have also examined the effect of individual gene knockout on the phenotype of improved ethanol tolerance to determine whether the up-regulated genes acted individually or as an ensemble [15, 17]. Their results shown that the knockout of most of these target genes resulted in loss of the improved phenotype, suggesting each gene was insufficient to confer ethanol tolerance individually. Therefore, it is assumed that the products of the up-regulated genes activated by the mutant Spt15 are necessary components of an interconnected network for the phenotype of ethanol tolerance, although some redundancy may exist [15].

## Conclusions

Ethanol tolerance and production of *K. marxianus* was improved by engineering TATA-binding protein Spt15. A single amino acid substitution (K31E) of TATA-binding protein Spt15 was able to bring differential expression of hundreds of genes in *K. marxianus*, leading to improvement in ethanol tolerance and production. RNA-Seq-based transcriptomic analysis revealed cellular transcription profile changes resulting from Spt15-M2: Spt15-M2 caused changes in transcriptional level of most of the genes in the central carbon metabolism network; genes associated with amino acid transport, long-chain fatty acid biosynthesis and MAPK signaling pathway were up-regulated, while genes related to ribosome biogenesis, translation and protein synthesis were down-regulated. Five candidate genes (*GAP1*, *GNP1*, *FAR1*, *STE2* and *TEC1*), which were found to be up-regulated in M2 strain, were overexpressed for a gain-of-function assay. However, the overexpression of no single gene helped improve ethanol tolerance as *SPT15*-M2 did, indicating that the DEGs acted as an interconnected network for the phenotype of ethanol tolerance. This work demonstrates that ethanol tolerance of *K. marxianus* can be improved by engineering its TATA-binding protein Spt15. This method can also be used for the improvement of other complex phenotypes in *K. marxianus* (such thermotolerance, oxidative stress tolerance, acetic acid tolerance, etc.) that have not been fully understood and are difficult to engineer using conventional methods of metabolic engineering. In addition to Spt15, other global transcription-related proteins such as transcription initiation factor TFIID subunit 10 (Taf10) and RNA polymerase II subunit Rpb7, or stress-related transcription factors such as Hsf1 or Msn2/4 can also be potential candidates to be engineered in the future.

## Methods

### Strains, plasmids and media

*Escherichia coli* TOP10 (Tiangen, Beijing, China) was used as a cloning host for DNA cloning and plasmid propagation. *Kluyveromyces marxianus* DMKU3-1042 (purchased from NITE Biological Resource Center) was used for ethanol tolerance experiments. The centromere plasmid pKmLP2 was constructed based on pAUR123 (Takara, Japan), with *AUR1*-*C* replaced by the G418-resistant gene *KanMX6*, *ARS1* and *CEN4* replaced by *K. marxianus CEN/ARS*, which has *ARS1* and centromeric functions. *E. coli* was grown in LB medium (1% tryptone, 0.5% yeast extract, 1% NaCl) containing 100 μg/mL ampicillin. *K. marxianus* was grown in YPD medium (1% yeast extract, 2% peptone and 2% glucose), with 200 μg/mL G418 sulfate added for strains transformed with pKmLP2-based vectors. Fermentation medium (FM) (20 g/L peptone, 20 g/L yeast extract, 200 g/L glucose, 0.6 g/L (NH4)_2_SO_4_, 0.15 g/L KH_2_PO_4_) was used for batch ethanol fermentation experiments.

### DNA manipulation

The genomic DNA of *K. marxianus* was isolated with EZNA^®^ Yeast DNA Kit (Omega Bio-tek, Doraville, CA, USA), following the supplier’s protocol. The *SPT15*-*GFP* co-expression vector was constructed using the method described by Szymczak-Workman et al. [69] (Additional file 1: Fig. S1). First the fragment containing a Kozak sequence, a *Sma*I site, *SPT15*, a GSG linker and the 5′ region of P2A was amplified with the oligos KmSPT15-F and KmSPT15-R as primers and genome DNA of *K. marxianus* as template. The fragment that contains the 3′ region of P2A, GFP gene and an *Xho*I site was amplified with oligos GFP-F and GFP-R. Then the resulting fragments were connected in a final overlap PCR with oligos KmSPT15-F and GFP-R as primers. The connected fragment *SPT15*-*P2A*-*GFP* was then cloned into pKmLP2 after digested with restriction enzymes *Sma*I and *Xho*I. The resulting plasmid was sequence verified to ensure the correct sequence of *SPT15*. All the primers used in this study were listed in Additional file 7.

### Construction of mutagenesis library

Mutagenesis primers (MUT-F and MUT-R) were designed based on the sequence of pKmLP2, with restriction sites of *Kpn*I and *Kpn*2I in each primer, respectively. Mutagenesis of *SPT15* was performed using error-prone PCR-based GeneMorph II Random Mutagenesis Kit (Agilent Technologies, Santa Clara, CA, USA). Then the pooled PCR products were purified using EasyPure PCR Purification Kit (TransGen Biotech, Beijing, China) and digested overnight at 37 °C using *Kpn*I and *Kpn*2I (ThermoFisher Scientific, Waltham, MA, USA). The vector pKmLP2-*SPT15* was also digested with the same restriction enzymes and the pKmLP2 backbone was purified using EasyPure Quick Gel Extraction Kit (TransGen Biotech, Beijing, China) after agarose gel electrophoresis. Then the purified PCR products were ligated into the pKmLP2 backbone using T4 DNA ligase (New England Biolabs, Beverly, MA, USA), followed by transformation into competent TOP10. The colonies were counted and the library size was determined to be 10^5^. Colonies were then pooled using sterile LB medium and a sterile cell scraper to create a liquid library of the cells. Pooled plasmids containing *SPT15* mutants were extracted using TIANprep Mini Plasmid Kit (Tiangen, Beijing, China) from *E. coli* and then transformed into *K. marxianus* DMKU3-1042 by electroporation. Details in electroporation *K. marxianus* are described in Additional file 1. The mutation frequency of the random mutagenesis library was determined by sequencing 20 randomly picked clones.

### Evaluation of P2A cleavage efficiency

The efficiency of P2A cleavage in *K. marxianus* was examined using semiquantitative western blotting analysis. Wild-type DMKU3-1042, the strain harboring *SPT15*-*P2A*-*GFP* cassette and the random mutagenesis library were grown overnight, collected and subjected to protein extraction, SDS-PAGE and western blotting. Protein extraction, SDS-PAGE and western blotting were conducted with reference to a previous study [70]. β-actin was monitored as a loading control. Anti-GFP mouse monoclonal antibody (Epsilon, Beijing, China) was used as primary antibody to monitor GFP and the uncleaved fusion of Spt15-P2A-GFP, while anti-β-actin mouse monoclonal antibody (Epsilon, Beijing, China) was used as primary antibody to monitor β-actin. HRP-conjugated goat anti-mouse IgG(H + L) (Epsilon, Beijing, China) was used as secondary antibody. The efficiency of P2A cleavage was calculated by dividing signal intensity value of GFP by the sum of the signal intensity values of GFP and the uncleaved fusion of Spt15-P2A-GFP.

### Mutant screening and identification

The random mutagenesis library was cultured in 200 mL YPD medium supplemented with elevated ethanol concentration [from 2 to 4% (v/v)] at 45 °C. After five successive subcultures, the enriched cell culture was diluted and spread onto YPD plates. Individual colonies were randomly picked for plasmid extraction using Zymoprep II kit (Zymo Research, Orange, CA, USA). The fragments containing *SPT15* mutants were amplified from the extracted plasmids using oligos SEQ-F and SEQ-R as primers. Then *SPT15* mutants were sequenced using the oligo SEQ-F as primer.

### Mutant growth under ethanol stress

Overnight cell cultures grown at 30 °C with shaking at 200 rpm were diluted with YPD medium containing 0, 4, 6% ethanol (v/v) to reach an initial OD_600_ (optical density at 600 nm) of ~ 0.20. These cell suspensions were aliquoted in triplicates into a sterile 96-well plate and incubated at 42 °C in a Tecan Infinite M200 Pro plate reader (Tecan Group Ltd., Männedorf, Switzerland) until stationary phase was reached. No higher temperature was applied due to the limit of instrument. The OD_600_ values in each well were automatically recorded at intervals of 60 min. Before each measurement, the 96-well plate was automatically shaken for 90 s to homogenize the samples.

To quantitatively investigate the growth curves, the growth curve data were fitted with logistic model [71, 72]:

$$y = A_{2} + \frac{{A_{1} - A_{2} }}{{1 + \left( {\frac{x}{{x_{0} }}} \right)^{p} }},$$

where *A*_1_, *A*_2_, *x*_0_ and *p* are parameters of logistic model.

Then the first-order derivative functions of OD_600_-time functions were calculated to study the growth speed variations.

For spotting test, 2 μL cell suspensions of each strain with OD_600_ of ~ 0.20 and serial dilutions of 10^−1^–10^−3^ were spotted onto YPD agar medium containing 0, 4 and 5% ethanol (v/v) and then incubated at 45 °C for 2 days.

### Batch ethanol fermentation

Batch fermentation experiments were conducted in sealed 100-mL serum bottles in triplicate at 45 °C. Samples were taken at intervals of 12 h for high-performance liquid chromatography (HPLC) analysis. Details in batch fermentation experiments and HPLC-based measurements of fermentation substrates and products are described in our previous study [8]. Intracellular content of pyruvate was measured using a pyruvate assay kit (BC2205, Solarbio, Beijing, China) following the manufacturer’s instructions.

### RNA-Seq-based transcriptomic analysis

To reveal the mechanisms of the ethanol tolerance conferred by the Spt15p-M2 mutant, the transcriptional profiles of M2 and control strain fermenting at 45 °C were investigated using RNA-Seq-based transcriptomic analysis with three biological replicates. Total RNA was extracted from the 20-h samples as described before [8] and then sent to Novogene Bioinformatics Technology (Beijing, China) for further quality and quantity evaluation, cDNA library preparation, and sequencing. We first compared the gene expression profiles between M2 and control strain to find the differentially expressed genes (DEGs) using DESeq R package [73]. The resulting *P* values were adjusted using the Benjamin and Hochberg’s approach for controlling the false discovery rate. Genes with adjusted *P* values (*P*_adj_) less than 0.05 and log_2_ (fold change) values greater than 1 were assigned as differentially expressed. Gene Ontology (GO) and Kyoto Encyclopedia of Gene and Genomics (KEGG) enrichment analyses of DEGs were implemented by the gene function classification tool of DAVID Bioinformatics Resource 6.8 (https://david.ncifcrf.gov/gene2gene.jsp) [74, 75]; GO and KEGG terms with *P* values less than 0.05 were considered significantly enriched. The interaction networks of DEGs were obtained using the STRING v10.5 database (http://string-db.org/) [76]. As there has been no *K. marxianus* data in the database, we chose *Saccharomyces cerevisiae* as the background.

## Additional files

**Additional file 1.** Additional methods and results.

**Additional file 2.** Logistic fitting results.

**Additional file 3.** RPKM values and fold changes of genes in the central carbon metabolic network.

**Additional file 4.** Differentially expressed genes for M2 vs *SPT15*.

**Additional file 5.** GO enrichment of differentially expressed genes for M2 vs *SPT15*.

**Additional file 6.** KEGG enrichment of differentially expressed genes for M2 vs *SPT15*.

**Additional file 7.** Primers used in this study.

## Abbreviations

ASSF: advanced solid-state fermentation
DEG: differentially expressed gene
EIC: ethanol inhibition concentration
GFP: green fluorescent protein
GO: gene ontology
gTME: global transcription machinery engineering
KEGG: Kyoto Encyclopedia of Gene and Genomics
NADH: nicotinamide adenine dinucleotide
NADPH: nicotinamide adenine dinucleotide phosphate
PCR: polymerase chain reaction
ROS: reactive oxygen species
PPP: pentose phosphate pathway
RNA-Seq: ribonucleic acid (RNA) sequencing
RPKM: reads per kilobase per million mapped reads
SSF: solid-state fermentation
TBP: TATA-binding protein
YPD: yeast extract, peptone and dextrose
