# Supplementary material for: Engineering TATA-binding protein Spt15 to improve ethanol tolerance and production in Kluyveromyces marxianus
Source: Biotechnol Biofuels. 2018 Jul 24;11:207. doi: 10.1186/s13068-018-1206-9 (PMC6058363; doi:10.1186/s13068-018-1206-9)
Supplement: Supplementary file 1 — Additional file 1. Additional methods and results. [file 13068_2018_1206_MOESM1_ESM.docx]

Additional Information

**Engineering TATA-binding protein Spt15 to improve ethanol tolerance and production in *Kluyveromyces marxianus***

**Authors:** Pengsong Li^†,*^, Xiaofen Fu^†^, Shizhong Li^*^ and Lei Zhang

**Affiliation:**

Institute of New Energy Technology, MOST-USDA Joint Research Center for Biofuels, Beijing Engineering Research Center for Biofuels, Tsinghua University, Beijing 100084, China

^†^Contributed equally

^*^Correspondence: [szli@mail.tsinghua.edu.cn](mailto:szli@mail.tsinghua.edu.cn) (S. Li); [lipengsong@mail.tsinghua.edu.cn](mailto:lipengsong@mail.tsinghua.edu.cn) (P. Li)

Email addresses of all the authors:

Pengsong Li: [lipengsong@mail.tsinghua.edu.cn](mailto:lipengsong@mail.tsinghua.edu.cn)

Xiaofen Fu: [dexter.dexter@163.com](mailto:dexter.dexter@163.com)

Shizhong Li: [szli@mail.tsinghua.edu.cn](mailto:szli@mail.tsinghua.edu.cn)

Lei Zhang: leizhangxny@tsinghua.edu.cn

**Electroporation of *Kluyveromyces marxianus***

An overnight culture of *Kluyveromyces marxianus* DMKU 3-1042 was harvested by centrifugation and washed twice with 1/10 initial volume of sterile water. Then the cells were resuspended in 1/10 initial volume of electroporation buffer (0.1 M LiAc, 0.1 M dithiothreitol, 0.6 M sorbitol, 0.01 M Tris-HCl pH 7.5) and incubated at room temperature for 30 min. The mixture was centrifuged and was washed with 1/10 initial volume of 1 M sorbitol, followed by being resuspended in 1/200 initial volume of 1 M sorbitol. Then 50 μl cell suspension was mixed with 5 μl pooled plasmids in a 2 mm electroporation cuvette (Bio-Rad, Hercules, CA, USA) and the electroporation was conducted using Gene Pulser Xcell electroporation system (Bio-Rad, Hercules, CA, USA). The electroporation condition was set as follows: voltage 1500 V, capacitance 25 μF, resistance 200 Ω. After electroporation, 1 ml 1 M sorbitol solution was added and the cell suspension was incubated at 30 ℃ for 1 h. Then 1 ml fresh YPD media was added and the cell suspension was incubated under shaking at 30 ℃ for another 1 h. The cell suspension was then plated on a YPD agar plate containing 200 μg/ml G418 sulfate followed by incubation at 30 ℃ for 72 h.

**
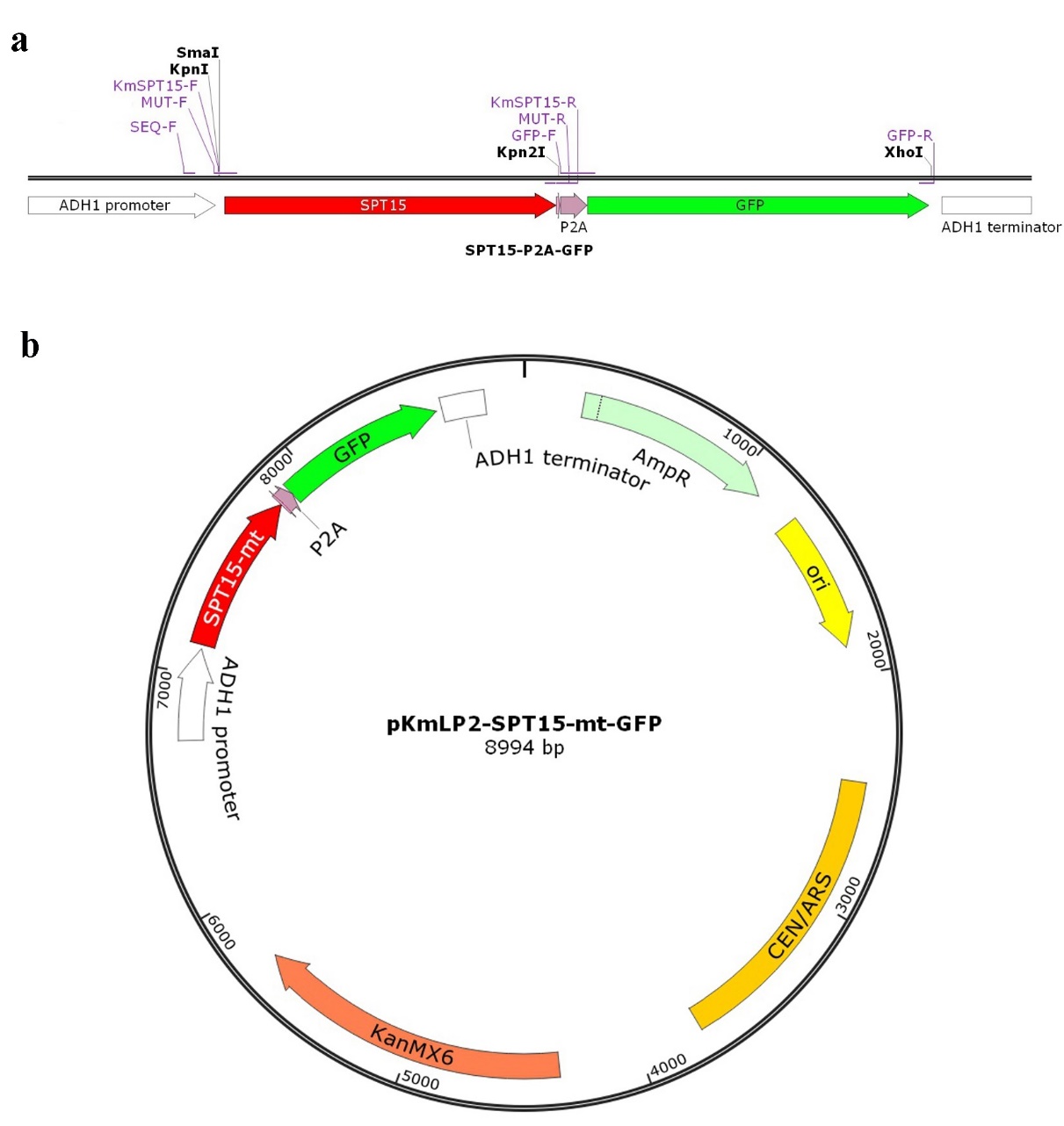
**

**Fig. S1 a.** The diagram of *SPT15-P2A-GFP* co-expression cassette. **b.** The map of *SPT15-mt-P2A-GFP* co-expression plasmid pKmLP2-SPT15-mt-GFP. *SPT15-mt*: mutants of *SPT15*; P2A: 2A peptide derived from porcine teschovirus-1; *GFP*: green fluorescent protein gene; *AmpR*: ampicillin-resistance gene; ori: pUC origin of replication; *CEN/ARS*: centromere and autonomous replicating sequence; *KanMX6*: G418-resistance gene


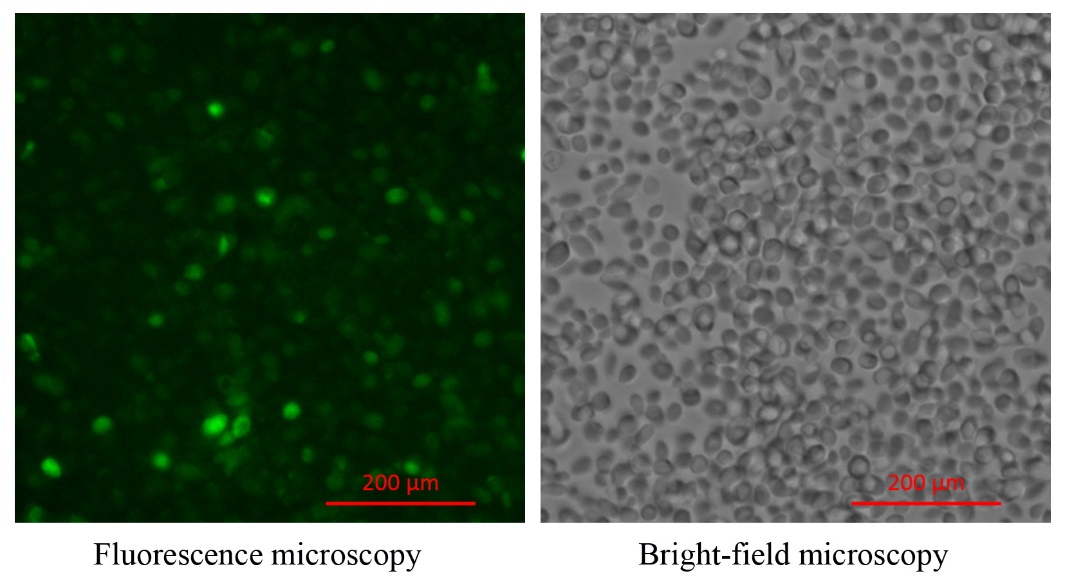


**Fig. S2** Fluorescence microscopy of the random mutagenesis library of *K. marxianus* in this study

**
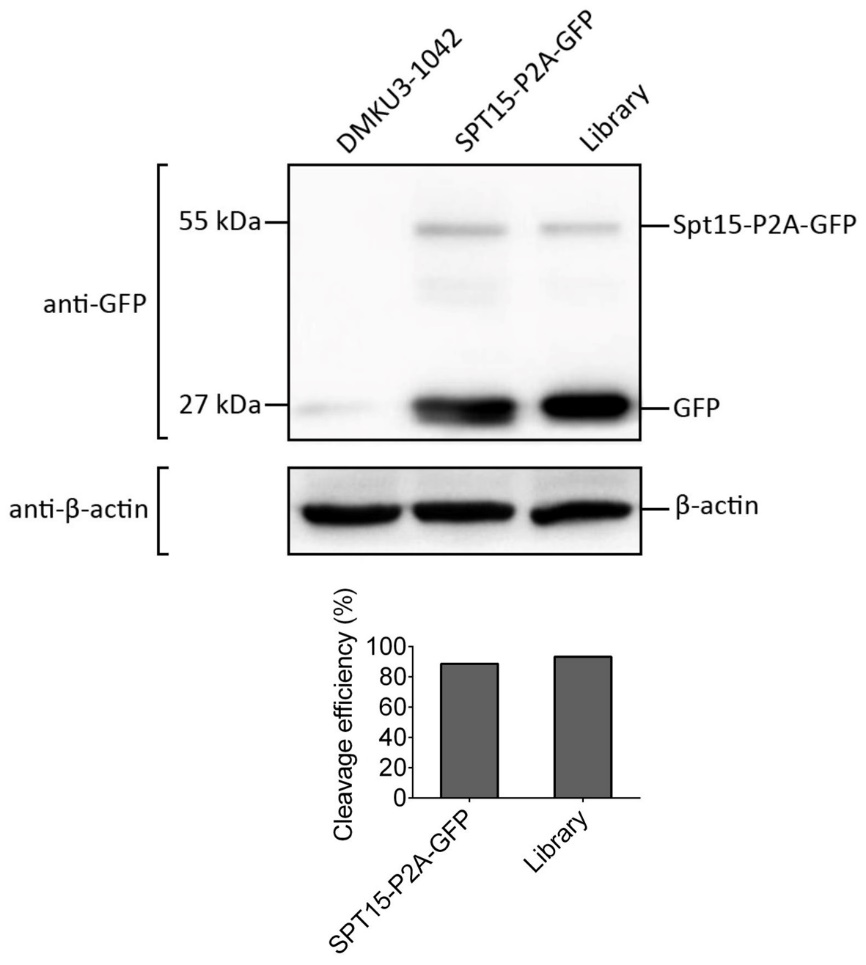
**

**Fig. S3** Semiquantitative Western blotting analysis to examine the efficiency of P2A cleavage in *K. marxianus*. Wild-type DMKU3-1042, strain harboring *SPT15-P2A-GFP* cassette and the random mutagenesis library were grown overnight, collected and subjected to protein extraction, SDS-PAGE and Western blotting. β-actin was monitored as a loading control. The efficiency of P2A cleavage was calculated by dividing signal intensity value of GFP by the sum of the signal intensity values of GFP and the uncleaved fusion of Spt15-P2A-GFP.


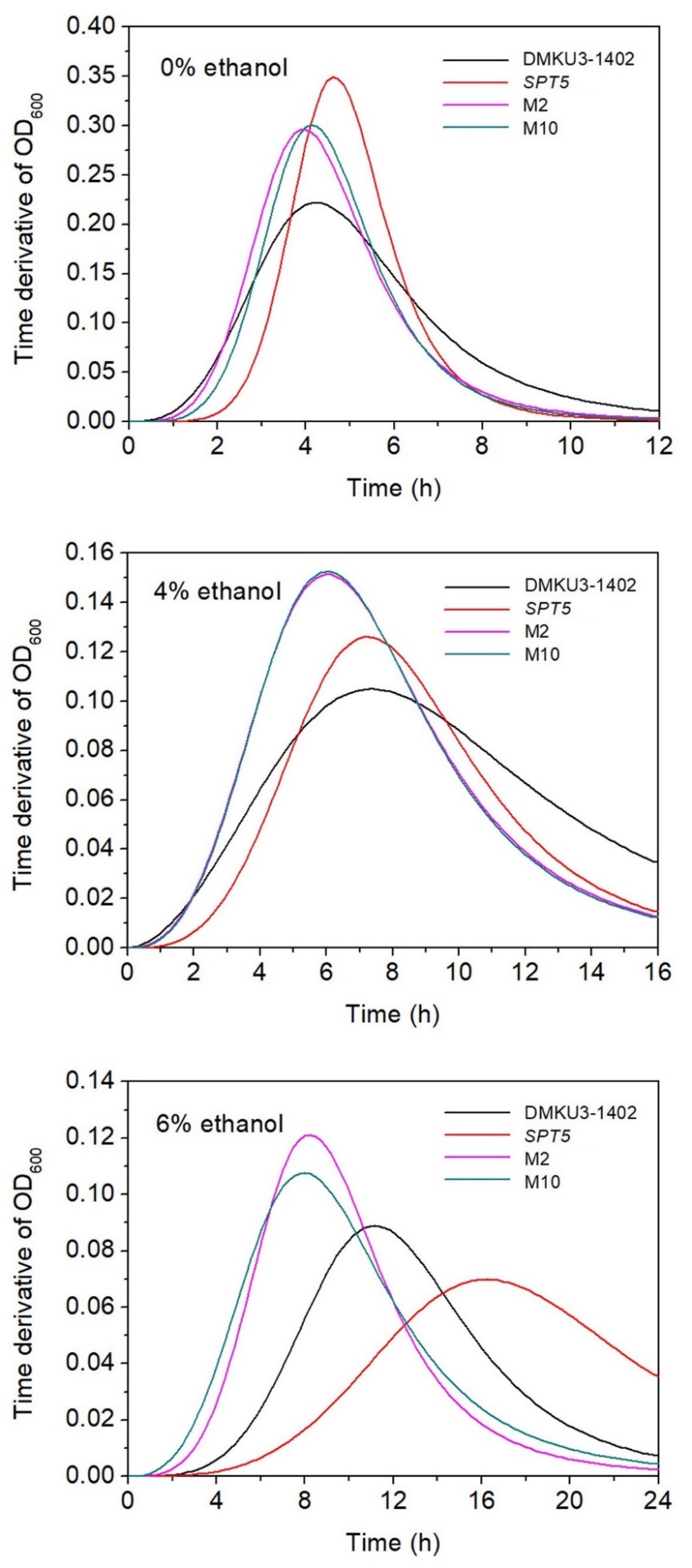


**Fig. S4** The first-order derivative curves of the growth curves of DMKU3-1402, *SPT15*, M2 and M10 at the presence of 0%, 4% and 6% ethanol at 45 ℃ (*SPT15* stands for the strain overexpressing wild-type *SPT15*)


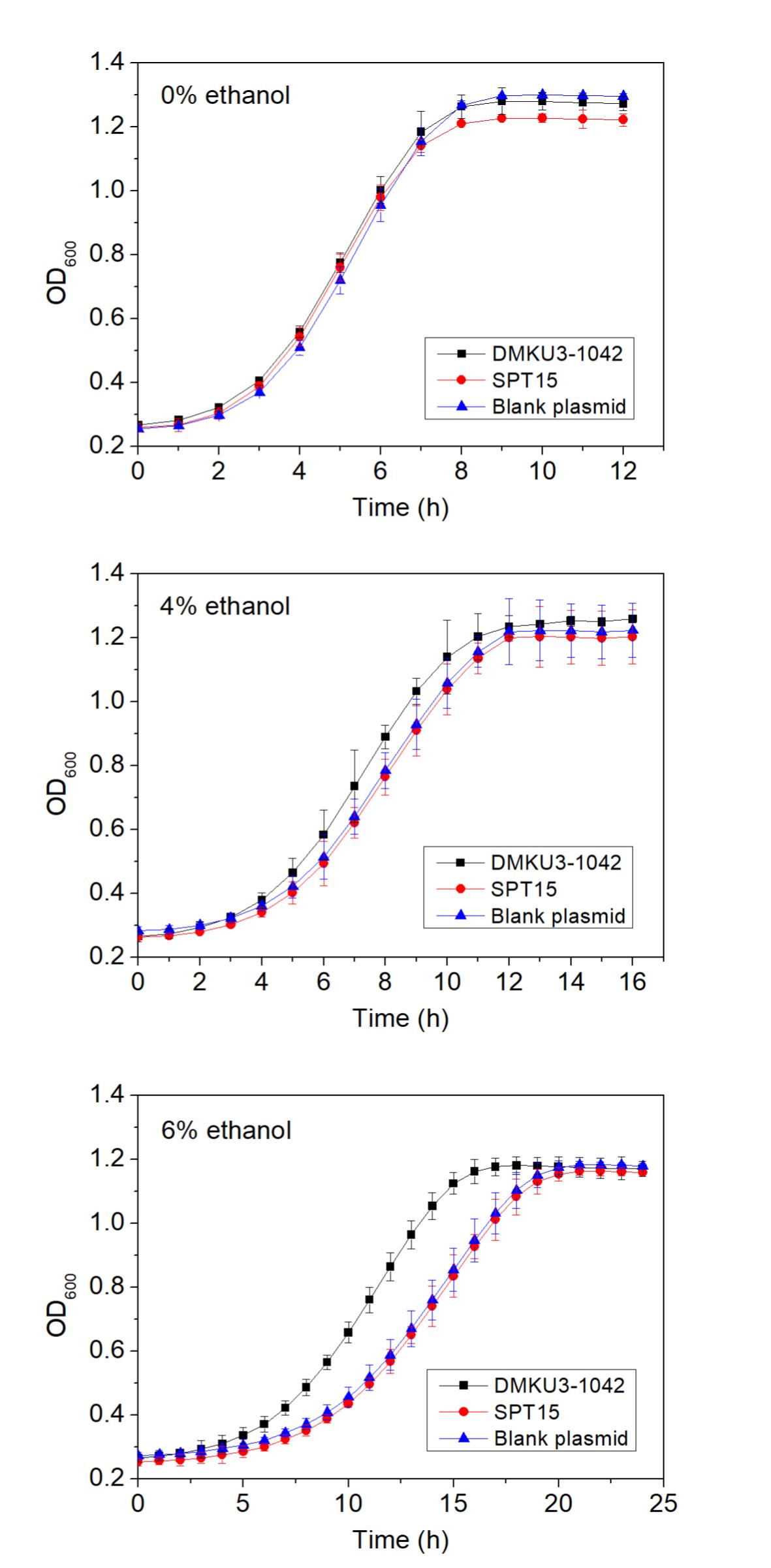


**Fig. S5** Growth curve assay to assess plasmid burden. Overnight cultures were diluted with YPD medium to reach an initial OD_600_ of 0.20. These cell suspensions were aliquoted in triplicate into a sterile 96-well plate with 200 μl in each well and incubated at 42 °C in a microplate reader to measure the growth curves. *SPT15*: strain overexpressing wild-type *SPT15*; blank plasmid: strain harboring plasmid pKmLP2. Values are means and standard deviations (n = 3)


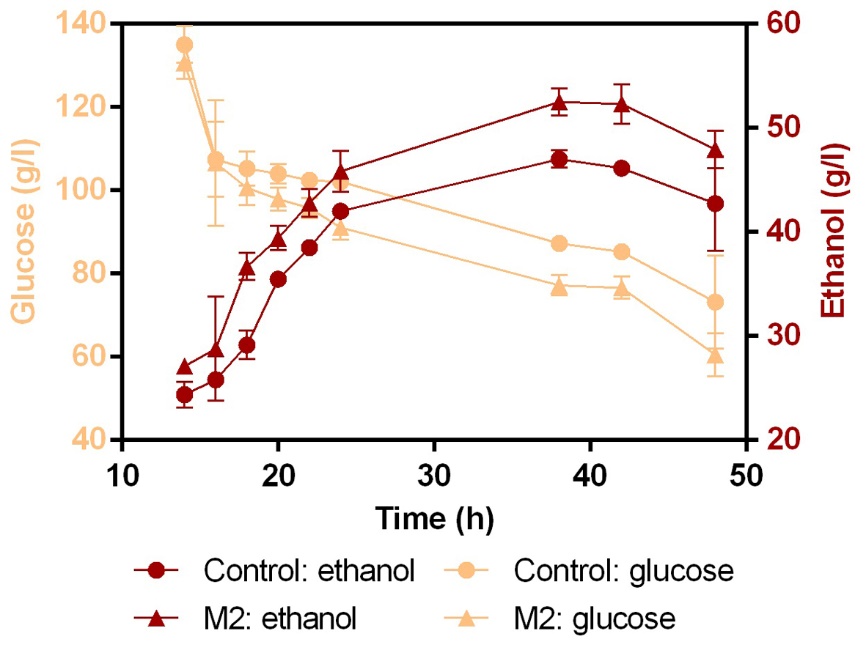


**Fig. S6** Concentrations of ethanol and residual glucose during ethanol fermentation. Data were collected from batch fermentation experiments conducted in sealed 100-ml serum bottles at 45 °C with three biological replicates. Re-constructed yeast strains without P2A-GFP were used. M2: strain expressing the *SPT15*-M2 mutant; control: strain overexpressing wild-type *SPT15*. Values are means and standard deviations (n = 3)


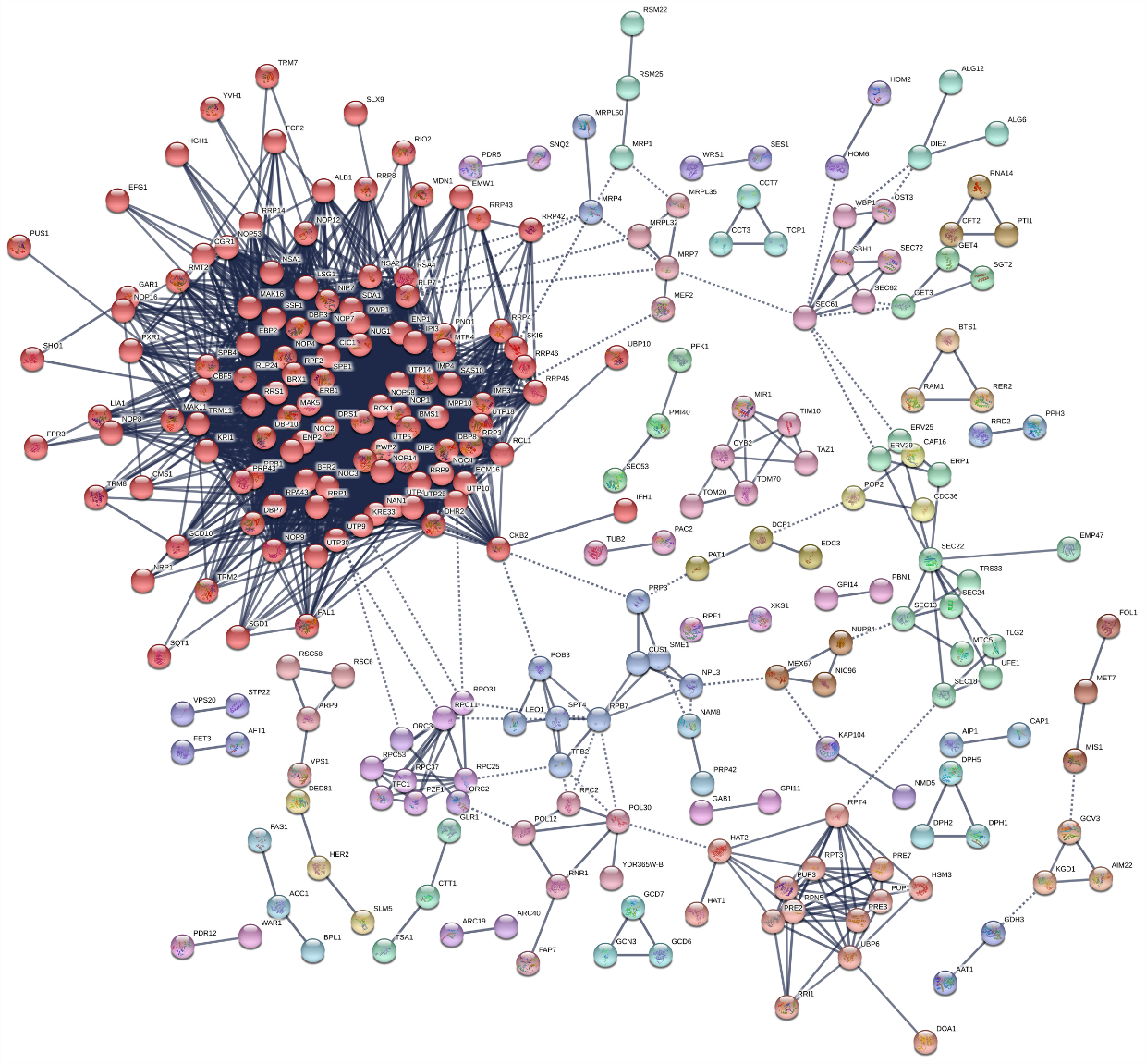


**Fig. S7** Interaction network of differentially expressed genes (DEGs) for M2 vs *SPT15* (score > 0.9). The network is clustered using MCL method with an inflation parameter of 2.0. Interactions are indicated by edges, with thicker edges having stronger associations. *SPT15* stands for the strain overexpressing wild-type *SPT15*

**
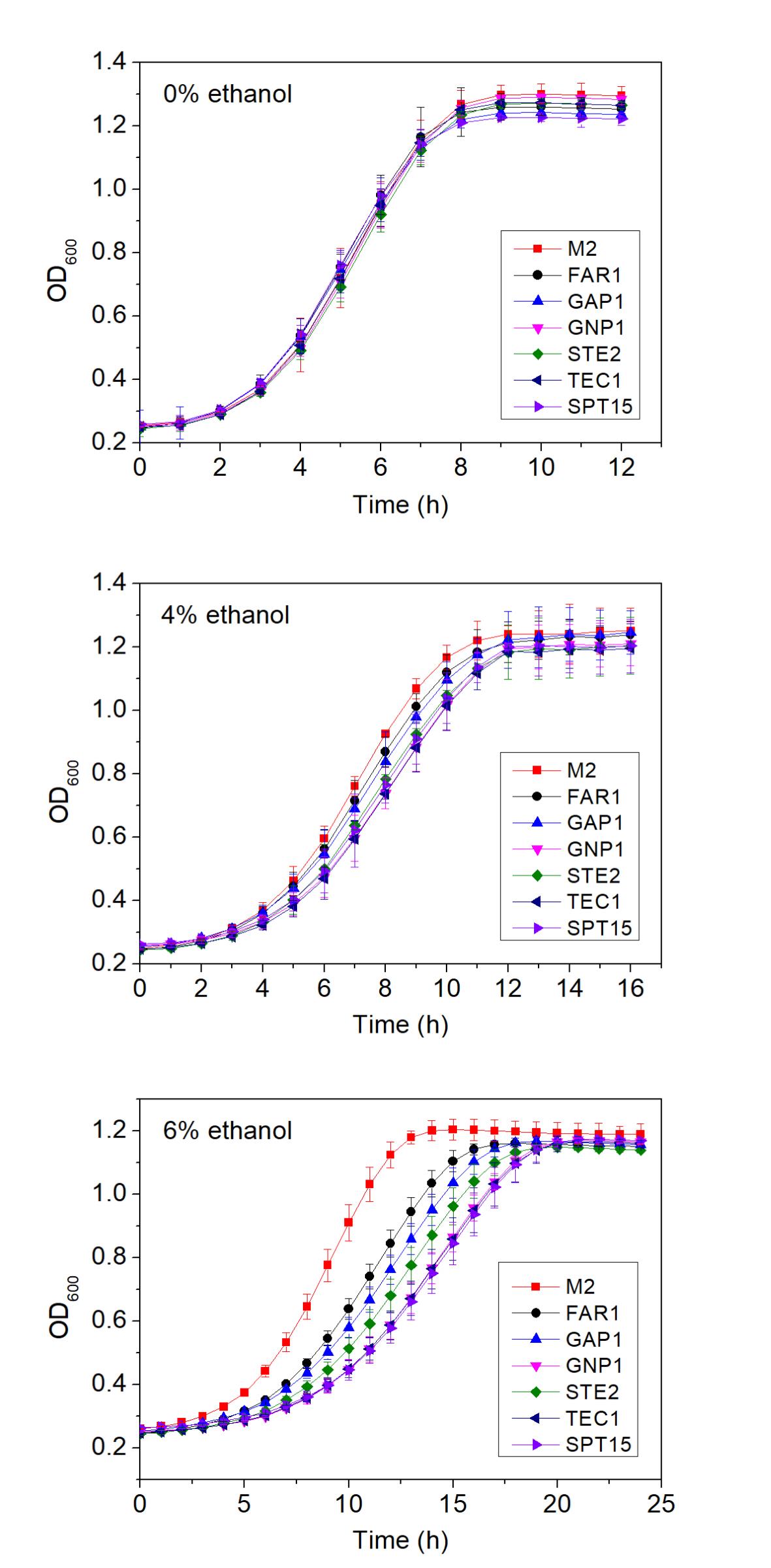
**

**Fig. S8** Growth curve assay to explore whether overexpression of an individual gene can lead to improved ethanol tolerance. Overnight cultures of strains overexpressing the *SPT15*-M2 mutant, *FAR1*, *GAP1*, *GNP1*, *STE2* and *TEC1* were diluted with YPD medium to reach an initial OD_600_ of 0.20. These cell suspensions were aliquoted in triplicate into a sterile 96-well plate with 200 μl in each well and incubated at 42 °C in a microplate reader to measure the growth curves. Values are means and standard deviations (n = 3)

**Table S1** Mutation statistics

| **Clone** | **Number of nucleotide change** |
| --- | --- |
| Clone 1 | 1 |
| Clone 2 | 0 |
| Clone 3 | 0 |
| Clone 4 | 4 |
| Clone 5 | 1 |
| Clone 6 | 0 |
| Clone 7 | 3 |
| Clone 8 | 3 |
| Clone 9 | 3 |
| Clone 10 | 2 |
| Clone 11 | 1 |
| Clone 12 | 3 |
| Clone 13 | 6 |
| Clone 14 | 2 |
| Clone 15 | 3 |
| Clone 16 | 3 |
| Clone 17 | 4 |
| Clone 18 | 1 |
| Clone 19 | 3 |
| Clone 20 | 3 |
| **Total** | 46 |
| **Average** | 2.3 |
